# Differential Susceptibility of Male Versus Female Laboratory Mice to *Anaplasma phagocytophilum* Infection

**DOI:** 10.3390/tropicalmed3030078

**Published:** 2018-07-23

**Authors:** Waheeda A. Naimi, Ryan S. Green, Chelsea L. Cockburn, Jason A. Carlyon

**Affiliations:** Department of Microbiology and Immunology, Virginia Commonwealth University Medical Center, School of Medicine, Richmond, VA 23298, USA; naimiw@mymail.vcu.edu (W.A.N.); greenrs@mymail.vcu.edu (R.S.G.); cockburnc@vcu.edu (C.L.C.)

**Keywords:** anaplasmosis, anaplasmataceae, mouse model, sex as a biological variable, morula, intracellular bacteria, gender differences to infection

## Abstract

Human granulocytic anaplasmosis (HGA) is a debilitating, non-specific febrile illness caused by the granulocytotropic obligate intracellular bacterium called *Anaplasma phagocytophilum*. Surveillance studies indicate a higher prevalence of HGA in male versus female patients. Whether this discrepancy correlates with differential susceptibility of males and females to *A. phagocytophilum* infection is unknown. Laboratory mice have long been used to study granulocytic anaplasmosis. Yet, sex as a biological variable (SABV) in this model has not been evaluated. In this paper, groups of male and female C57Bl/6 mice that had been infected with *A. phagocytophilum* were assessed for the bacterial DNA load in the peripheral blood, the percentage of neutrophils harboring bacterial inclusions called morulae, and splenomegaly. Infected male mice exhibited as much as a 1.85-fold increase in the number of infected neutrophils, which is up to a 1.88-fold increase in the *A. phagocytophilum* DNA load, and a significant increase in spleen size when compared to infected female mice. The propensity of male mice to develop a higher level of *A. phagocytophilum* infection is relevant for studies utilizing the mouse model. This stresses the importance of including SABV and aligns with the observed higher incidence of infection in male versus female patients.

## 1. Introduction

Sex as a biological variable (SABV) is an important criterion to include in vertebrate animal studies. As part of its approach to increase rigor and reproducibility, the United States (USA) National Institutes of Health recently changed its guidelines to ensure that its funded vertebrate animal studies address SABV. As many as 37% of published preclinical animal studies and 9% of published cell culture studies may have been affected by these new guidelines [1]. Biological differences between males and females have been shown to affect infection rates and variable gene transcription of multiple pathogens including *Mycobacterium tuberculosis* [2,3], *Legionella* spp. [4], and *Coxiella burnetii* [5]. Even though infections caused by bacteria in the order Rickettsiales have led to the loss of millions of lives throughout history, have impacted wars, and continue to threaten global health today [6,7,8,9,10,11,12,13], no study to date has addressed SABV for any rickettsial organism.

*Anaplasma phagocytophilum* is a tick-transmitted obligate intracellular bacterium of the order Rickettsiales that preferentially infects granulocytes and bone marrow progenitor cells to cause human granulocytic anaplasmosis (HGA). The emerging infection typically presents as a non-specific febrile illness that is self-limiting in otherwise healthy individuals. In the elderly, the immunocompromised, and, in some instances, when proper antibiotic therapy is delayed, HGA can result in potentially deadly sequelae including rhabdomyolysis, septic shock, and pneumonia [14]. The first HGA case was documented in Minnesota, USA in 1994 [15]. Since then, the disease has been increasingly reported in the USA, Europe, and Asia [14]. From the year 2000, when HGA was reported to the USA Centers for Disease Control (CDC), up to 2016, the number of annually reported cases rose from 350 to 4151, which represents a 1186% increase [16]. Seroepidemiologic data indicate that its incidence may be several-fold higher in some endemic areas [17,18]. Notably, it has been reported that male HGA patients outnumber female HGA patients three to one [19].

The mouse model has been utilized to study *A. phagocytophilum* infection in vivo for more than 20 years. Infected mice develop laboratory markers that occur during HGA. One of these markers shows that neutrophils harboring intravacuolar *A. phagocytophilum* colonies, called morulae, can be detected in peripheral blood smears using light microscopy. *A. phagocytophilum* infected mice also develop splenomegaly [20,21,22,23,24,25,26]. Whether male or female mice exhibit differential susceptibility to infection with the bacterium has yet to be explored. This fact has led to the current study. In this paper, we demonstrate that male mice exhibit significantly higher peripheral blood bacterial burdens and splenomegaly than female mice after infection with *A. phagocytophilum*.

## 2. Materials and Methods

### 2.1. Cultivation of Uninfected and A. phagocytophilum Infected Cell Lines

Uninfected and *A. phagocytophilum* (NCH-1 strain) infected human promyelocytic HL-60 cells, CCL-240 (American Type Culture Collection (ATCC), Manassas, VA, USA) were cultured as described previously [27].

### 2.2. Literature Search

To determine if prior publications that utilized the mouse model of granulocytic anaplasmosis examined the influence of SABV on *A. phagocytophilum* infection, queries of PubMed (https://www.ncbi.nlm.nih.gov/m/pubmed) were performed using combinations of the following keywords or keyword combinations: *A. phagocytophilum*, granulocytic anaplasmosis, granulocytic ehrlichiosis, human granulocytic anaplasmosis, human granulocytic ehrlichiosis, agent of human granulocytic ehrlichiosis, mouse, murine, and in vivo. The time period examined was 1980 to present. The searches returned a total of 61 different publications.

### 2.3. Infection of C57/Bl6J Mice

Seven-week-old female and male C57Bl/6J mice (Jackson Laboratories, Bar Harbor, ME, USA) were housed in animal biosafety level-2 laboratories prior to and during all experiments. Mice were intraperitoneally inoculated with 1 × 10^8^ *A. phagocytophilum* organisms that had been recovered following sonication of infected HL-60 cells and differential centrifugation, which is a method that specifically isolates the infectious dense-core but not the non-infectious reticulate cell form [28]. Sterile PBS was used as a mock inoculum to inject negative control animals. Blood was collected from the tail vein on days 0, 4, 8, 12, 16, 21, and/or 28. On day 28, the mice were euthanized, blood was collected via cardiac puncture, and spleens were harvested. All animal research was performed under the approval of the Institutional Animal Care and Use Committee at Virginia Commonwealth University (Protocol Number AM10220).

### 2.4. Evaluation of A. phagocytophilum Infection

Peripheral blood smears were fixed and stained with FisherBrand Hema 3 solutions (ThermoFisher, Waltham, MA, USA) and examined by light microscopy for the presence of neutrophils with morulae. Three blood smears were examined per time point per mouse in order for the percentage of infected neutrophils to be determined from a total of at least 300 neutrophils. DNA was isolated from heparin-treated blood using the DNeasy Blood and Tissue Kit (Qiagen, Valencia, CA, USA). Fifty nanograms of DNA were subjected to quantitative PCR (qPCR) using primers targeting *A. phagocytophilum* 16S rDNA and mouse *β-actin* [29], SsoFast EvaGreen Supermix (Biorad, Hercules, CA, USA), and the CFX384 Detection System (Biorad). Thermal cycling conditions consisted of an initial denaturation step of 98 °C for 2 min and was followed by 40 cycles at 98 °C for 5 s and 60 °C for 30 s. The relative 16 S rDNA levels were normalized to that of *β-actin* using the 2^−ΔΔCT^ method [30]. To assess for splenomegaly, spleen-to-body weight ratios were determined on day 28.

### 2.5. Statistical Analysis

Statistical analyses were performed using the Prism 5.0 software package (Graphpad, San Diego, CA, USA). Two-way analysis of variance (ANOVA) with the Sidek’s post-hoc test was used to test for a significant difference among the groups. A paired student’s *t*-test was used to test for statistical significance between paired data. Statistical significance was set at *p* values of <0.05.

## 3. Results

### 3.1. A. phagocytophilum Infected Male Mice Have Higher Peripheral Blood Bacterial DNA Levels than Infected Female Mice

**Figure 1:**
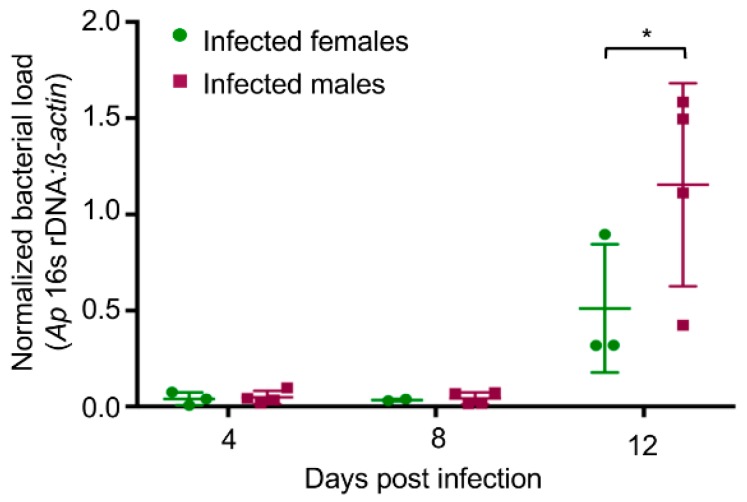
*A. phagocytophilum* infected male mice exhibit higher peripheral *A. phagocytophilum* DNA levels than female mice. Male and female C57Bl/6 mice were intraperitoneally injected with *A. phagocytophilum* organisms. DNA samples isolated from heparin-treated blood recovered on days 4, 8, and 12 and were then subjected to qPCR using gene-specific primers. Relative *A. phagocytophilum* 16S rDNA-to-murine *β-actin* DNA levels for each mouse generated using the 2^−ΔΔCT^ method along with their standard deviations are presented. Statistically significant (* p < 0.05) values are indicated.

An examination of the literature revealed 61 publications that used the mouse model of granulocytic anaplasmosis. Of the 61 reports, 31 did not disclose the gender, 13 used only females, 9 used only males, 6 included both sexes, 1 used females for one group and males for another, and 1 used females for the experimental group but male and females for the control group [20,21,22,23,25,26,31,32,33,34,35,36,37,38,39,40,41,42,43,44,45,46,47,48,49,50,51,52,53,54,55,56,57,58,59,60,61,62,63,64,65,66,67,68,69,70,71,72,73,74,75,76,77,78,79,80,81,82,83] (Table 1). None of the six studies that included males and females examined for a correlation between sex and differential susceptibility to *A. phagocytophilum* infection [25,39,41,62,65,73]. We sought to determine if such a correlation exists. Following inoculation, the *A. phagocytophilum* peripheral blood burden in immunocompetent wild-type mice tends to peak by day 12 and subsides thereafter to undetectable or near undetectable levels by days 21 to 28 [23,26,38,45,52,58,60,64,68,71,73,75,78,81,84]. C57Bl/6 mice are commonly used for studying *A. phagocytophilum* infection [26,34,36,38,39,40,41,52,61,62,65,68,70,73,75,76,78,84,85]. Male and female C57Bl/6 mice were intraperitoneally inoculated with host cell-free *A. phagocytophilum* organisms. DNA was isolated from blood obtained on days 4, 8, and 12 and subjected to qPCR using primers targeting *A. phagocytophilum* 16S rDNA and mouse *β-actin*. On day 12, the relative bacterial load in the peripheral blood of male mice exhibited a statistically significant 1.88-fold increase relative to bacterial load in the peripheral blood of female mice (Figure 1).

### 3.2. A. phagocytophilum Infected Male Mice Exhibit Higher Percentages of Neutrophils Harboring Morulae and Splenomegaly Compared to Infected Female Mice

**Figure 2:**
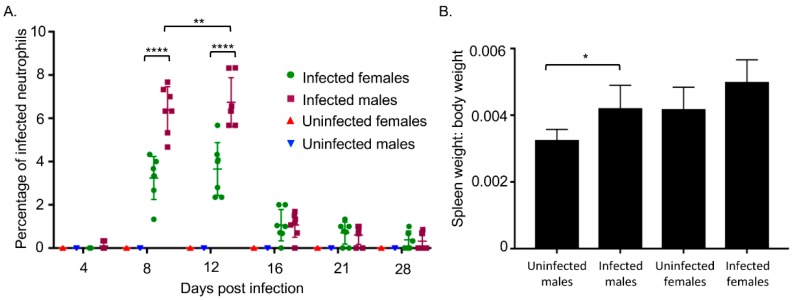
*A. phagocytophilum* infected male mice have higher percentages of peripheral blood neutrophils harboring morulae and greater degrees of splenomegaly than infected female mice. Male and female C57Bl/6 mice were intraperitoneally injected with *A. phagocytophilum* bacteria. (**A**) Peripheral blood smears were microscopically examined for neutrophils that contained morulae. Each dot corresponds to the mean percentage of infected neutrophils determined from counting a total of at least 300 neutrophils from three separate blood smears per mouse. Error bars correspond to the mean ± standard deviation of the percentages determined for all seven mice per group. Data are representative of two experiments with similar results; (**B**) On day 28, the mice were euthanized, the spleens were harvested, and the spleen-to-body weight ratios were calculated. Data are the mean ± standard deviation of the ratios determined from seven mice per group. Statistically significant (* p < 0.05, ** p < 0.01, **** p < 0.0001 values are indicated.

The infection experiment was repeated except that each sex group consisted of seven mice. To address the possibility that *A. phagocytophilum* might achieve its highest peripheral load in female mice at a later time point than day 12, blood was drawn on days 4, 8, 12, 16, 21, and 28. In addition, since qPCR measures the DNA load and, therefore, cannot distinguish DNA derived from live versus dead bacteria, blood smears were generated and examined for the presence of neutrophils harboring morulae. Consistent with the qPCR data, both sexes had the highest mean percentage ± SD of neutrophils with morulae on day 12 (Figure 2A). However, the percentages of infected neutrophils in female mice never became as high as that observed in male mice, which were significantly 1.97-fold and 1.85-fold greater on days 8 and 12, respectively. Little to no infected peripheral blood neutrophils were detectable for either sex by day 28. Overall, these data indicate that, while the kinetics of *A. phagocytophilum* infection do not differ between male and female mice over the course of infection, the bacterium achieves a significantly higher load in the peripheral blood in male mice. On day 28, the mice were euthanized, spleens harvested, and their spleen-to-body weight ratios determined. The mean spleen-to-body weight ratio of infected to uninfected male mice increased significantly while the mean spleen-to-body weight ratio of infected to uninfected female mice did not (Figure 2B).

## 4. Discussion

This study demonstrates for the first time that *A. phagocytophilum* achieves a higher peripheral blood load and causes a greater degree of splenomegaly in male versus female mice using the C57Bl/6 strain, which is the most-commonly used murine model of granulocytic anaplasmosis. These results are consistent with a higher case incidence in males among HGA patients [19]. The biological basis for this difference is unclear. Given that *A. phagocytophilum* incorporates cholesterol into its cell wall [86], replicates better in the presence of cholesterol [61,87,88], and specifically exploits the low-density lipoprotein (LDL) uptake pathway [88], it is reasonable to speculate that the better infection the bacterium exhibits in males could be at least partially linked to the fact that men tend to have higher LDL-cholesterol levels than women [89]. A report using apolipoprotein E-deficient mice and mice fed high cholesterol diets conspicuously demonstrated that high blood cholesterol levels facilitate *A. phagocytophilum* infection. However, that study, which only assessed the relevance of cholesterol to *A. phagocytophilum* infection, exclusively utilized male mice [61]. It would be worth revisiting this study using males and females. Another possible gender-based difference that could account for the reduced permissiveness of females to *A. phagocytophilum* is that female mice and humans tend to produce higher levels of IFNγ and exhibit more robust innate and adaptive immune responses than males. This difference translates to better clearance, reduced susceptibility, and lower disease incidence of viral and fungal infections in female mice [90,91,92,93]. An IFNγ dominated immune response is critical for clearing *A. phagocytophilum* infection [59].

Whether a similar sex bias for better *A. phagocytophilum* infection exists in other laboratory mouse strains remains to be determined. Due to the importance of including SABV as a criterion when designing vertebrate animal experiments unless a sex bias in the model exists, the differential susceptibility of male versus female mice to *A. phagocytophilum* infection demonstrated in this paper should be kept in mind for future experiments using the C57Bl/6 strain.

## Figures and Tables

**Table 1:** Published studies using the mouse model of granulocytic anaplasmosis.

References	Usage of Female and/or Male Mice
[20,21,22,26,31,32,34,35,36,38,40,42,45,46,47,48,49,51,54,55,56,59,60,63,66,72,74,75,78,79,80]	Not disclosed
[23,43,52,53,58,64,67,68,69,70,71,81,82]	Females
[33,37,44,50,57,61,77,83,84]	Males
[25,39,41,62,65,73]	Males and females
[85]	Females used for experimental group and males used for control group
[76]	Females used for experimental group and males and females used for controls
