# Correction: Naimi, W.A., et al. Differential Susceptibility of Male versus Female Laboratory Mice to *Anaplasma phagocytophilum* Infection. *Trop. Med. Infect. Dis.* 2018, *3*, 78

**DOI:** 10.3390/tropicalmed4010051

**Published:** 2019-03-23

**Authors:** Waheeda A. Naimi, Ryan S. Green, Chelsea L. Cockburn, Jason A. Carlyon

**Affiliations:** Department of Microbiology and Immunology, Virginia Commonwealth University Medical Center, School of Medicine, Richmond, VA 23298, USA; naimiw@mymail.vcu.edu (W.A.N.); greenrs@mymail.vcu.edu (R.S.G.); cockburnc@vcu.edu (C.L.C.)

The authors wish to make the following corrections to this paper [1]. In the context of Table 1 and Results Section 3.1, two references were misinterpreted with regards to the sexes of mice used in those studies. As such, they were miscategorized in Table 1 and incorrectly referenced in Results Section 3.1. These mistakes have been resolved. The authors would like to apologize for any inconvenience caused to the readers by these changes.

We have recently been made aware by Dr. Mustafa Akkoyunlu (Food and Drug Administration) of two errors that we made in interpreting whether or not groups of mice used in studies by Dr. Akkoyunlu and other researchers were sex-matched. The two specific studies that we misinterpreted are references numbers [76,85]. The third paragraph of the said Results Section currently reads as follows (please note that the underlined text is what will be edited):

*3.1. A. phagocytophilum Infected Male Mice Have Higher Peripheral Blood Bacterial DNA Levels than Infected Female Mice*

An examination of the literature revealed 61 publications that used the mouse model of granulocytic anaplasmosis. Of the 61 reports, 31 did not disclose the gender, 13 used only females, 9 used only males, 6 included both sexes, 1 used females for one group and males for another, and 1 used females for the experimental group but male and females for the control group [20–23,25,26,31–83] (Table 1). None of the six studies that included males and females examined for a correlation between sex and differential susceptibility to *A. phagocytophilum* infection [25,39,41,62,65,73]. We sought to determine if such a correlation exists. Following inoculation, the *A. phagocytophilum* peripheral blood burden in immunocompetent wild-type mice tends to peak by day 12 and subsides thereafter to undetectable or near undetectable levels by days 21 to 28 [23,26,38,45,52,58,60,64,68,71,73,75,78,81,84]. C57Bl/6 mice are commonly used for studying *A. phagocytophilum* infection [26,34,36,38–41,52,61,62,65,68,70,73,75,76,78,84,85]. Male and female C57Bl/6 mice were intraperitoneally inoculated with host cell-free *A. phagocytophilum* organisms. DNA was isolated from blood obtained on days 4, 8, and 12 and subjected to qPCR using primers targeting *A. phagocytophilum* 16S rDNA and mouse *β-actin*. On day 12, the relative bacterial load in the peripheral blood of male mice exhibited a statistically significant 1.88-fold increase relative to bacterial load in the peripheral blood of female mice (Figure 1).

To correct our error, we would like to make the following corrections:

*3.1. A. phagocytophilum-Infected Male Mice Have Higher Peripheral Blood Bacterial DNA Levels than Infected Female Mice*

An examination of the literature revealed 61 publications that used the mouse model of granulocytic anaplasmosis. Of the 61 reports, 32 did not disclose the gender, 14 used only females, 10 used only males, and 6 included both sexes [20–23,25,26,31–85] (Table 1). None of the six studies that included males and females examined for a correlation between sex and differential susceptibility to *A. phagocytophilum* infection [25,39,41,62,65,73,85]. We sought to determine if such a correlation exists. Following inoculation, the *A. phagocytophilum* peripheral blood burden in immunocompetent wild-type mice tends to peak by day 12 and subsides thereafter to undetectable or near-undetectable levels by days 21 to 28 [23,26,38,45,52,58,60,64,68,71,73,75,78,81,84]. C57Bl/6 mice are commonly used for studying *A. phagocytophilum* infection [26,34,36,38–41,52,61,62,65,68,70,73,75,76,78,84,85]. Male and female C57Bl/6 mice were intraperitoneally inoculated with host-cell-free *A. phagocytophilum* organisms. DNA was isolated from blood obtained on days 4, 8, and 12 and subjected to qPCR using primers targeting *A. phagocytophilum* 16S rDNA and mouse *β*-actin. On day 12, the relative bacterial load in the peripheral blood of male mice exhibited a statistically significant 1.88-fold increase relative to the bacterial load in the peripheral blood of female mice (Figure 1).

Change in Tables

Because the above misinterpreted references are cited in Table 1, we have adjusted Table 1 as follows, replace:
**References****Usage of Female and/or Male Mice**[20–22,26,31,32,34–36,38,40,42,45–49,51,54–56,59,60,63,66,72,74,75,78–80]Not disclosed[23,43,52,53,58,64,67–71,81,82]Females[33,37,44,50,57,61,77,83,84]Males[25,39,41,62,65,73]Males and females[85]Females used for experimental group and males used for control group[76]Females used for experimental group and males and females used for controls
with
**References****Usage of Female and/or Male Mice**[20–22,26,31,32,34–36,38,40,42,45-49,51,54–56,59,60,63,66,72,74–76,78–80]Not disclosed[23,43,52,53,58,64,67–71,81,82,84]Females[33,37,44,50,57,61,77,83–85]Males[25,39,41,62,65,73]Males and females
